# Intracranial pressure changes during early postoperative mobilization in patients with chronic subdural hematoma

**DOI:** 10.1007/s00701-025-06655-9

**Published:** 2025-09-01

**Authors:** Anders Schack, Markus Harboe Olsen, Jonathan Truels Hansen, Anna Søgaard Magnussen, Ida Møller Larsen, Helene Ravnholt Jensen, Marianne Juhler, Alexander Lilja-Cyron, Kåre Fugleholm, Thorbjørn Søren Rønn Jensen

**Affiliations:** 1 Department of Neurosurgery, Copenhagen University Hospital – Rigshospitalet, Inge Lehmanns vej 6, DK-2100 Copenhagen, Denmark; 2 Department of Neuroanaesthesiology, Neuroscience Centre, Copenhagen University Hospital – Rigshospitalet, Inge Lehmanns vej 6, DK-2100 Copenhagen, Denmark

**Keywords:** MeSH, Chronic subdural hematoma, Intracranial pressure, Postoperative care, Early ambulation, Mobilization

## Abstract

**Background:**

Passive subdural drainage is used to reduce the risk of chronic subdural hematoma (cSDH) recurrence and mortality. However, the effects of patient positioning on intracranial pressure (ICP) during passive drainage remain unclear.

**Objective:**

To examine how subdural drainage and patient positions influence postoperative ICP in cSDH patients with a subdural passive drainage system.

**Methods:**

This was a prospective, single-center observational cohort study. Eleven patients (mean age 78 years) underwent burr hole evacuation for cSDH with placement of a subdural drain connected to a bile bag system. An ICP probe was placed in the subdural space posteriorly via the same burr hole. During the first 48 h, ICP was measured in supine, 30° head elevation, and sitting positions before and after removal of the subdural drain. Given the small sample size, the study was a priori considered hypothesis‑generating; the width of confidence intervals and potential type II error are therefore emphasised throughout. Also, the associations between ICP and radiographic parameters (midline shift, hematoma size, pneumocephalus) were assessed.

**Results:**

When all positions were combined, mean ICP with the drain in situ was − 2.5 (− 4.8 to − 0.2) mmHg versus − 0.5 (− 2.9 to 1.9) mmHg after removal (*p* = 0.233). Position-specific differences after vs before removal were likewise small and non-significant: supine -1.9 (-7.8 to 4.1, *p* = 0.537), 30° -2.6 mmHg (-8.5 to 3.3, *p* = 0.378), sitting -3.6 (-9.8 to 2.6, *p* = 0.244) mmHg; mixed-effects modelling confirmed these findings.

Greater midline shift and hematoma volume were associated with higher ICP, whereas larger pneumocephalus volumes correlated with lower ICP.

**Conclusions:**

After burr-hole evacuation of cSDH, ICP remained within normal physiological limits across all head positions, both with the subdural drain in situ and after its routine removal at 24 h. Providing that the drainage height is adjusted to the pivot point for CSF pressure at shoulder level, mobilization—even to the upright position—did not provoke dangerously low ICP. These data, together with emerging randomised evidence, support early mobilisation after cSDH surgery; nonetheless, confirmation in adequately powered multicentre trials is required before firm practice recommendations can be issued.

**Supplementary Information:**

The online version contains supplementary material available at 10.1007/s00701-025-06655-9.

## Introduction

Chronic subdural hematoma (cSDH) is a frequent neurosurgical condition in elderly persons, characterized by a delayed presentation that often occurs weeks to months historically linked to a minor, sometimes unnoticed, head injury, with growing evidence suggesting that ongoing inflammation, cerebral atrophy, and microvascular fragility are central pathophysiological drivers [4, 8, 13]. Burr hole drainage is generally acknowledged as a standard treatment, but is associated with recurrence rates around 10–20%, underscoring the need for optimized postoperative management [9]. One aspect of this management is passive drainage via a bile bag, a technique employed after cSDH evacuation to allow fluid to drain without active suction. While this strategy is widely adopted in clinical practice [1, 10], there is limited understanding of how varying patient positions affect intracranial pressure (ICP), particularly under passive drainage conditions.

Previous bench-model investigations have demonstrated that passive drainage systems can generate measurable negative pressures—depending on factors such as fluid-column height and bag placement—even though they are considered “passive” [12]. However, the direct impact of upright mobilization and positioning on real-time ICP in patients with a cSDH has not been fully elucidated [12]. Early postoperative mobilization is often encouraged to reduce the risk of complications such as deep vein thrombosis and to promote a faster functional recovery, yet concerns persist that positional changes might destabilize ICP, especially in the context of a recent burr hole evacuation [20]. Furthermore, high-pressure gradients generated by closed-suction surgical drainage systems has been acknowlegded [19].

This observational study examines early postoperative ICP changes in patients with cSDH who have undergone burr hole drainage with a passive bile bag system. By measuring ICP at various time points and patient positions, ranging from supine to upright sitting, we aim to clarify whether mobilizing these patients in the initial postoperative period significantly influences ICP.

## Methods

### Study design and ethical approval

We conducted a single-center, prospective observational study at the Department of Neurosurgery, Copenhagen University Hospital – Rigshospitalet (Copenhagen, Denmark) to investigate changes during early mobilization following burr-hole evacuation of cSDH. The study was designed in accordance with a predefined protocol approved by the Regional Committee on Health Research Ethics and the Danish Data Protection Agency (H-21052143, 23 January 2023). Written informed consent was obtained from all participants.

### Participants

We screened consecutive patients aged 60 years or older who were admitted with a diagnosis of cSDH requiring surgical intervention (burr-hole evacuation) and who were able to provide an informed consent for participation in the study.

Baseline demographic and clinical data including midline shift measured on preoperative computed tomography (CT), hematoma volume, and comorbidities were recorded upon inclusion. Patients were followed for 90 days, including observation for late recurrences.

### Surgical procedure and drain placement

All procedures were standardized according to the Danish national guidelines and the DACSUHS standard procedure [10]. Based on the preoperative imaging a 13 mm burr hole was drilled at the apex of the hematoma. Before incising the dura, baseline ICP was obtained by inserting the ICP probe (sensor) through a red 18 G needle penetrating the dura mater into the hematoma, and the initial ICP was recorded using CODMAN® ICP EXPRESS® Monitor (Integra Lifesciences, Princeton, NJ, USA). Next, the dura mater was incised in a cross. The cSDH was gently evacuated by flushing the subdural space with sterile saline at body temperature until any residual hematoma fluid and debris were adequately cleared.

**Fig. 1:**
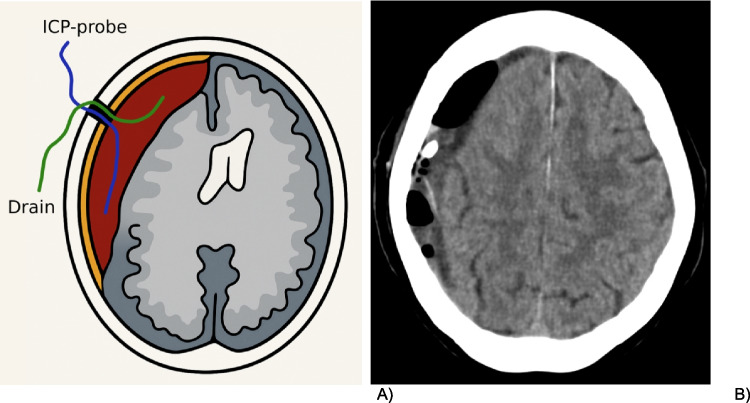
Placement of ICP-probe and subdural drain. **A** Schematic **B** Post-operative brain CT

Following complete irrigation, the Spiral Drain (Redax™, Poggio Rusco, Italy) was inserted into the subdural space in an anterior direction to allow continuous passive drainage of any residual fluid, while the sensor was positioned posteriorly in the same burr hole corridor to minimize measurement interference (Fig. 1).

The subdural drain was then connected externally at shoulder height fixed to skin to a bile bag drainage system without applying any active suction, allowing fluid to drain via passive gravitational flow. The surrounding scalp was closed in a standard layered fashion, leaving both the drain and ICP probe externalized.

### ICP Measurement schedule and data collection

Following surgery, patients were transferred to the Post Anesthesia Care Unit (PACU), where immediate ICP measurements were collected using the ICP monitor and stored in ICM + (Cambridge Enterprise, University of Cambridge, United Kingdom). Thereafter, an early mobilization protocol was implemented, progressing patients from supine to 30° head-of-bed elevation and ultimately to a sitting position, as tolerated[6]. Between measurement sessions, patients were encouraged to move freely to facilitate functional recovery.

ICP was continuously recorded during the first 48 postoperative hours. For standardized comparisons, discrete measurement points were defined as follows:

1. PACU, immediate postoperative
2. PACU, prior to ward transfer (within 6 h of the procedure)
3. 8–16 h postoperatively
4. 24 h postoperatively (just before drain removal)
5. 24 h postoperatively (immediately after drain removal)
6. 32–40 h postoperatively
7. 48 h postoperatively

At each of these time points, ICP values were recorded in three distinct patient positions:

- Supine (0° head elevation)
- 30° head elevation
- Sitting upright

Patients remained in each position for five minutes to allow the ICP to stabilize [16]. The time and mobilization details for each measurement were noted, and the continuous ICP record was subsequently reviewed around those intervals. Characteristic shifts in the ICP curve indicated when the patient changed position, and a stable plateau was identified in each posture. The mean ICP from this plateau was then calculated and recorded as the representative value for that position. This approach accounted for minor discrepancies between the recorded measurement time and the exact moment of repositioning, ensuring that the analyzed ICP values were both stable and physiologically meaningful in this observational setting.

A routine CT scan was performed 24 h after the initial procedure, while both the subdural drain and ICP probe were still in place to ensure correct placement of probe and drain. Midline shift, residual hematoma volume, and the presence/volume of pneumocephalus were quantitatively assessed on the CT scan.

### Outcomes

1. Mean ICP (mmHg) at each of the predefined postoperative time points and in each of the three patient positions (supine, 30° elevation, and sitting).
2. Changes in ICP before and after subdural drain removal
3. Relationship between ICP and radiographic findings (midline shift, hematoma size and post-operative pneumocephalus)

### Statistical analysis

Given the exploratory, within-subject nature of this pilot study, a cohort of 11 patients was deemed acceptable because guidance for pilot work recommends roughly 12 observations per group to yield sufficiently precise estimates of the mean and variance for planning future trials [2]. Continuous variables were summarized by mean and 95% confidence interval (CI) or median and interquartile range, depending on data distribution. Categorical variables were reported as counts and percentages.

Data distributions were assessed visually for normality; variables that conformed to normal assumptions were analyzed with parametric tests, whereas non-normal variables were analyzed with nonparametric alternatives. Paired comparisons (e.g., ICP differences across time points or positions within the same patient) used the paired t test. For between-group comparisons (e.g., ICP before vs. after drain removal across different patients), unpaired t tests were applied as appropriate.

To account for repeated measurements per patient, a mixed-effects linear regression model was fitted in sensitivity analyses, with patient ID as a random effect to capture within-subject correlation. Correlations between ICP and radiographic variables (midline shift, hematoma size, pneumocephalus) were investigated using Pearson’s correlation (for normally distributed data).

A *p*-value < 0.05 was considered statistically significant for all comparisons. No correction for multiplicity was applied, as this was an exploratory and hypothesis generating study. All analyses were conducted using R version 4.4.2 (R Core Team, Vienna, Austria).

## Results

A total of 11 patients (8 men, 73%) were included. The mean age was 78 years (95% CI: 76–80). The mean preoperative midline shift was 8.2 mm (95% CI: 5.9–10.5), and the mean hematoma volume was 139.7 cm^3^ (95% CI: 92.6–186.8). The mean pneumocephalus volume on the first postoperative CT scan was 23.9 cm^3^ (95% CI: 12.8–35.0). The opening ICP had a mean of 7.7 mmHg (95% CI: 5.4–10.0), with one patient missing baseline data.

Across all time points, the highest mean ICP occurred in the supine position (2.9 mmHg; 95% CI 0.1 to 5.7), followed by 30° elevation (− 1.6 mmHg; 95% CI − 4.4 to 1.1), and in sitting position (− 5.9 mmHg; 95% CI − 8.7 to − 3.1). The average difference between sitting and supine was 8.8 mmHg ([95% CI 7.2 to 10.4]; *p* < 0.001).

**Fig. 2:**
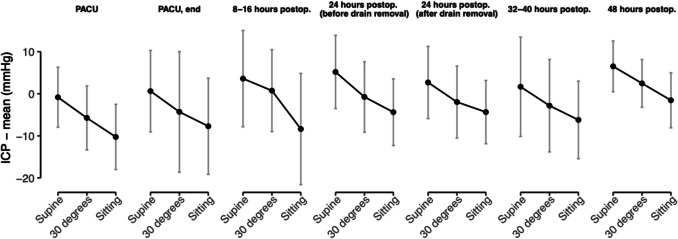
Mean intracranial pressure (ICP) readings (± standard deviation) over the first 48 h post-surgery, comparing supine, 30° head-elevated, and sitting positions. Measurements were taken in the post-anesthesia care unit (PACU), at the end of PACU stay, 8–16 h postoperatively, at 24 h (both before and after drain removal), 32–40 h, and 48 h

Figure 2 illustrates mean ICP values, along with standard variability, in the supine, 30° head-elevated, and sitting positions at each measurement point over the initial 48 h post-surgery. Overall, ICP readings typically ranged from about − 10 to + 5 mmHg, with a mild upward trend over time. Supine measurements consistently showed the highest ICP, followed by 30° elevation and then sitting. At the initial postoperative measurement in the PACU, ICP stayed below 0 mmHg across all positions, with several patients reaching values below − 10 mmHg during the first 16 h. Over time, mean ICP progressed from negative readings in the PACU to slightly positive by 48 h, with the following mean values (in mmHg) at each time point: PACU, − 5.62; 95% CI − 9.60 to − 1.63; end of PACU, − 3.75; 95% CI − 9.26 to 1.77; 8–16 h, − 1.24; 95% CI − 6.95 to 4.47; 24 h (before drain removal), 0.04; 95% CI − 4.43 to 4.50; 24 h (after drain removal), − 1.19; 95% CI − 5.46 to 3.09; 32–40 h, − 3.17; 95% CI − 8.10 to 1.76; and 48 h, 2.29; 95% CI − 0.96 to 5.55.

**Fig. 3:**
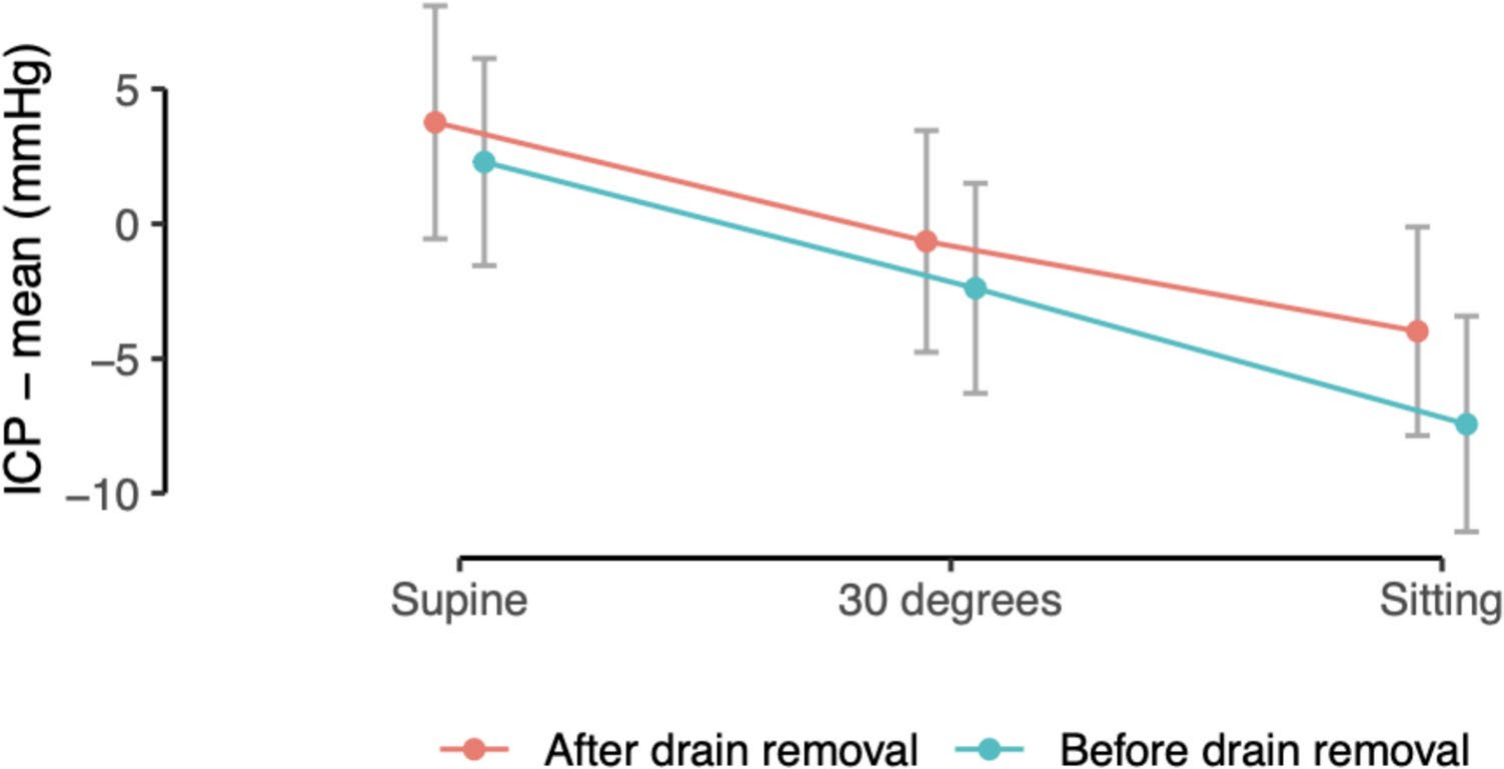
Mean intracranial pressure (ICP) values (± standard deviation) in supine, 30° head-elevated, and sitting positions, comparing measurements taken before (blue) versus after (red) subdural drain removal

Combining all positions, the mean ICP were nominally lower with drainage (− 2.5 mmHg [95% CI − 4.8 to − 0.2]) compared to ICP after drain removal (− 0.5 mmHg [− 2.9 to 1.9], p = 0.233; Fig. 3). The difference in mean ICP (after vs. before drain removal) was − 1.9 mmHg (95% CI − 7.8 to 4.1, p = 0.537) in the supine position, − 2.6 mmHg (− 8.5 to 3.3, p = 0.378) at 30°, and − 3.6 mmHg (− 9.8 to 2.6, p = 0.244) while sitting. A sensitivity analysis using mixed-effects linear regression similarly showed no significant effect of drain removal on ICP (estimated differences 0.30 mmHg, p = 0.866; − 0.57 mmHg, p = 0.739; and − 1.6 mmHg, p = 0.447 for supine, 30°, and sitting, respectively).

**Fig. 4:**
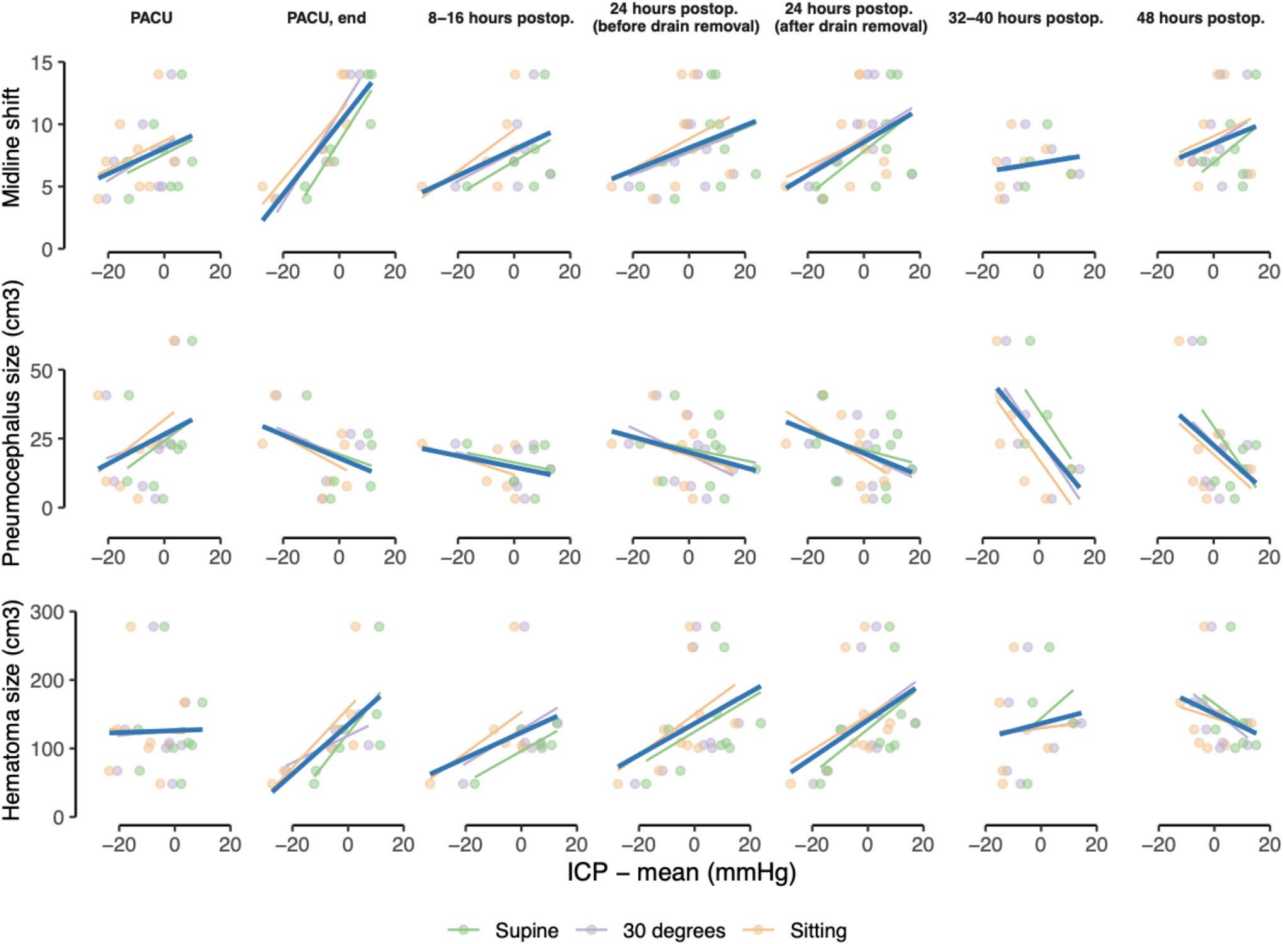
Scatter plots with regression lines showing the relationships between mean intracranial pressure (ICP) on the x-axis and midline shift (top row), postoperative pneumocephalus size (middle row), and preoperative hematoma size (bottom row) on the y-axis across the study’s time points. Colors represent measurements taken in supine (green), 30° head elevation (purple), and sitting (orange). Larger preoperative midline shifts and hematoma volumes were generally associated with higher ICP, whereas greater pneumocephalus volume showed an inverse correlation with ICP

Figure 4 illustrates the relationships between ICP and three key variables: midline shift (4a), postoperative pneumocephalus volume (4b), and preoperative hematoma size (4c). In panel (a), patients with larger midline shifts consistently showed higher ICP values across all positions and time points. A similar pattern emerged in panel (c), where greater hematoma size (often correlated with midline shift) was likewise associated with higher ICP. By contrast, panel (b) indicates an inverse relationship between ICP and postoperative pneumocephalus volume. Supplementary Fig. 1a-c demonstrates ICP values separated based on position.

### Clinical outcomes and adverse events

None of the patients required urgent reoperation, and there were no documented infections or other serious complications attributable to the ICP probe or the passive drainage system. All participants showed clinical improvement in neurological status by the final 48-h assessment, and none experienced an ICP crisis necessitating medical or surgical intervention. No patient experienced symptomatic recurrence within the 48‑hour observation window, and 90‑day clinical follow‑up confirmed that none of the eleven patients required re‑operation for recurrence. Nonetheless, the study was not powered nor designed for definitive long‑term recurrence assessment.

## Discussion

This study provides an in vivo presentation of ICP dynamics in patients undergoing burr hole evacuation of cSDH with a passive bile bag drainage system. Although bench experiments have demonstrated that passive drains can generate surprisingly high levels of negative pressure comparable to partially compressed active-suction devices, our clinical observations did not reveal sustained or harmful reductions in ICP in the early postoperative period. Despite theoretical concerns that an upright posture could accentuate siphoning and over-drainage, ICP in our cohort remained well within physiological limits. Normative and reference data place ICP at 1.0 mmHg (95% CI –5.9 to 8.3) in the upright position and 8.6 mmHg (95% CI 0.9 to 16.3) when supine [11, 14]. By comparison, we recorded a mean ICP of 2.9 mmHg (95% CI 0.1 to 5.7) in the supine position and –5.9 mmHg (95% CI –8.7 to –3.1) in the sitting/upright position—approximately 5 mmHg lower than the corresponding reference values.

Although mean differences were small and non‑significant, upper confidence limits approached values that could be clinically meaningful. The absence of statistical significance therefore likely reflects limited study power rather than true equivalence, and the observed trends might indeed reach significance in a larger, adequately powered cohort—an important direction for future research.

Marked siphoning can occur when a passive drain becomes fluid-filled, raising legitimate worries that sudden drops in ICP might promote tension pneumocephalus, venous re-bleeding, or impaired perfusion—especially in elderly brains with poor compliance [12]. Bench models, however, overstate the clinical hazard. Physiological autoregulation dampens rapid ICP swings; minor variations in bag height, intermittent air gaps, and incomplete column filling further blunt negative pressure; and the residual membranes plus the viscoelastic subdural/brain interface provide an additional buffer.

Our data, and others’ clinical series [1, 7, 15] show that passive drains rarely produce ICP shifts of clinical concern, even though their peak suction rivals that of partially compressed active systems [12]. Tension pneumocephalus or re-bleeding remains theoretical, more likely with fully fluid-loaded tubing, but real-world experience suggests that autoregulatory and compartmental elasticity limit these risks. Notably, closed-suction devices can generate still higher gradients [19], underscoring the need for observant drain management and vigilant monitoring rather than abandonment of passive drainage altogether.

It is also noteworthy that none of the patients in this study developed clinically significant complications such as re-bleeding, tension pneumocephalus, or hypotensive headaches that could be attributed to excessively low ICP. Interestingly, the observation that ICP remained low but stable during the early postoperative period—even with mobilization—is somewhat counterintuitive. Traditionally, it has been assumed that maintaining patients in flat bedrest would minimize negative pressure gradients and thus promote more effective brain re-expansion. However, our findings suggest that modest negative pressures generated by passive drainage and patient positioning may, paradoxically, facilitate brain re-expansion without adverse effects. Additionally, while Fig. 3 hints at an inverse association between the volume of postoperative pneumocephalus and mean ICP, it is still uncertain whether intracranial air lowers ICP by acting as a compressible buffer or whether a pre-existing low-pressure environment merely favors the ingress and persistence of air.

Clinical trial data align with this physiology. In the randomized GET-UP program, starting head-of-bed elevation and out-of-bed activity within 12 h of burr-hole evacuation cut early medical complications and produced a numerically lower re-operation rate than 48 h of strict recumbency; one-year follow-up showed an ≈18-percentage-point absolute gain in favorable GOSE scores with no excess recurrences [18, 20]. Taken together, the physiologic and trial evidence support routine early mobilization after cSDH surgery.

Ambulation in the early postoperative phase after cSDH evacuation raises a long‑standing concern that vertical posture could place traction on fragile bridging veins, and has recently been discussed [3, 5, 17, 21]. Our high‑resolution ICP recordings demonstrate that across all standard postures—including full sitting—both before and after drain removal, pressure fluctuations were minor and rapidly reversible. By converting a theoretical safety concern into objective physiological data, the study offers mechanistic evidence that complements outcome‑based research and clarifies the intrinsic safety profile of early mobilization.

Despite the reassuring findings, certain limitations warrant caution. The study’s small sample size and its confinement to a single center limit the generalizability of the findings. Furthermore, the 48-h postoperative observation window may be insufficient to detect complications that develop later, such as delayed fluid re-accumulation or late-onset neurological deterioration, which could have important clinical implications. Although none of the eleven patients had a 90‑day recurrence, the study was not powered to establish definitive long‑term recurrence rates. Additionally, while continuous ICP monitoring offers a detailed physiologic picture, the observational design limits any causal inferences regarding specific interventions to reduce negative pressure (e.g., restricting how low the drainage bag is placed). Further constraints include (i) lack of morphological stratification, (ii) exclusive use of a single burr‑hole technique, and (iii) the reliance on passive rather than active drainage—all of which may limit external validity.

In conclusion, during the first 48 h postoperatively patients undergoing burr hole evacuation of a cSDH have notable negative subdural pressure with a downward trend during mobilization. However, ICP measured during mobilization with a subdural drain in place is similar to previously published reference values for normal ICP we therefore support early mobilization for this patient group. Given the observational design and limited power, these findings should be interpreted as supportive rather than definitive; multicentre randomised studies are warranted.

## Supplementary Information

Below is the link to the electronic supplementary material.

ESM 1 (DOCX 368 KB)

## Data Availability

De-identified participant-level data, statistical code and an annotated study protocol will be available upon request, honoured by the corresponding author within two weeks.
