# Supplementary material for: Intracranial pressure changes during early postoperative mobilization in patients with chronic subdural hematoma
Source: Acta Neurochir (Wien). 2025 Sep 1;167(1):234. doi: 10.1007/s00701-025-06655-9 (PMC12402039; doi:10.1007/s00701-025-06655-9)
Supplement: Supplementary file 1 — ESM 1 (DOCX 368 KB) [file 701_2025_6655_MOESM1_ESM.docx]

**Supplementary:**


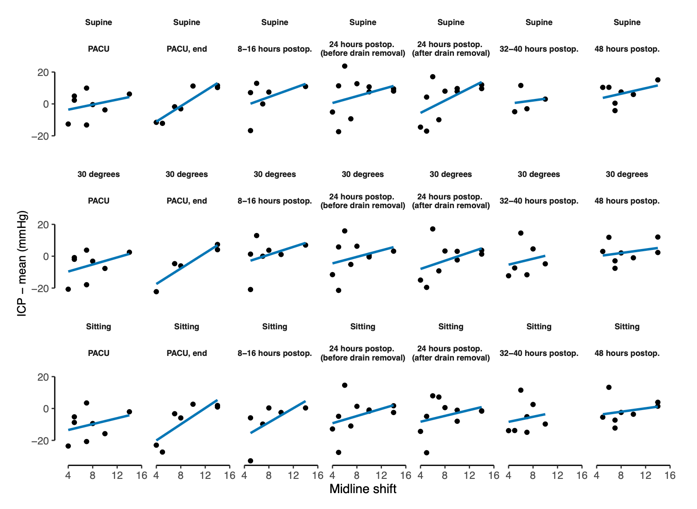


a)


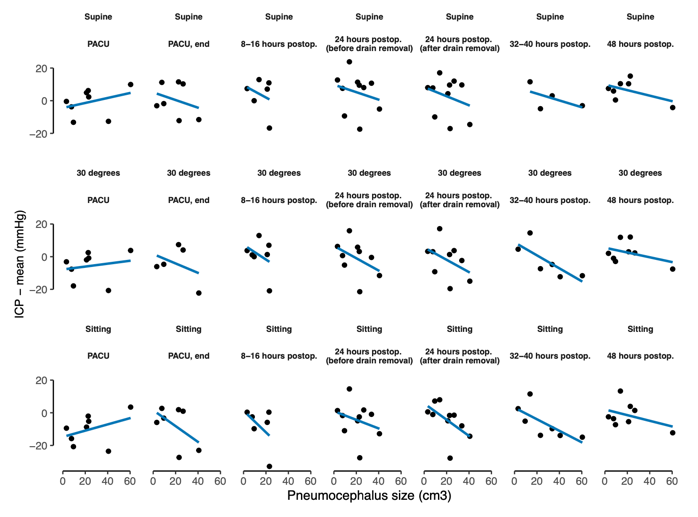


b)


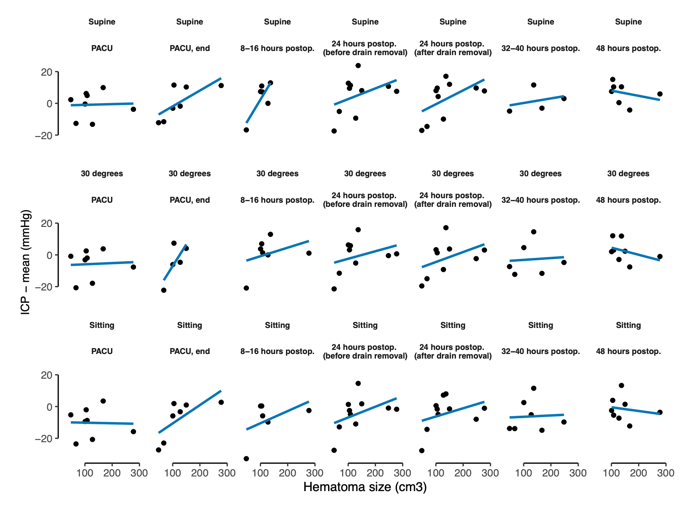


c)

**Supplementary Fig. 1 a–c:** Scatter plots depicting the relationships between mean intracranial pressure (ICP) and radiographic variables: (a) midline shift, (b) pneumocephalus volume, and (c) preoperative hematoma size—stratified by each position (supine, 30° elevation, and sitting). Each panel highlights how these variables correlate with ICP under different positional conditions throughout the study period.
